# Interspecific comparison of allometry between body weight and chest girth in domestic bovids

**DOI:** 10.1038/s41598-017-04976-z

**Published:** 2017-07-06

**Authors:** Hiroki Anzai, Kazato Oishi, Hajime Kumagai, Eiji Hosoi, Yoshitaka Nakanishi, Hiroyuki Hirooka

**Affiliations:** 1 Graduate School of Agriculture, Kyoto University, Kyoto, 606-8502 Japan; 2 Faculty of Agriculture, Yamaguchi University, Yamaguchi, 753-8515 Japan; 3 Faculty of Agriculture, Kagoshima University, Kagoshima, 890-8580 Japan

## Abstract

The sizes of body parts often co-vary through exponential scaling, known as allometry. The evolution of allometry is central to the generation of morphological diversity. To make inferences regarding the evolved responses in allometry to natural and artificial selection, we compared allometric parameters (slope and intercept) among seven species and breeds of domestic bovids using cross-sectional ontogenetic data and attempted to interpret the differences in these parameters. The allometric slopes were not different among some species, whereas those between breeds within species were, indicating that the slopes were typically invariant but could be changed under strong, specific selection. With the exception of yak, the differences in the intercept independent of the slopes (the alternative intercept) among species might better correspond to their divergence times than the differences in allometric slope, and the remarkably higher alternative intercept found in yaks can be explained by their unique morphological evolution. These findings provide evidence that differences in the alternative intercept can retain traces of the phylogenetic changes derived from differentiation and evolution.

## Introduction

The shape of an animal is a fundamental feature of its overall design, and the variations in shape among animals are due to differences in the proportions of various body parts relative to the body as a whole. The size of a body part is strongly correlated with the size of other parts or the whole body through exponential scaling, which is known as allometry. The allometric relationship follows a power law, *Y* = *aX* ^*b*^, where *Y* represents a body part, *X* represents another part or the whole, and *a* and *b* are constants^1, 2^. On a logarithmic scale, the relationship becomes linear, with an intercept of log(*a*) and slope of *b*. The slope, *b*, is the “constant differential growth rate” or “allometric coefficient” and its constancy indicates that the ratio between the rates of growth in *Y* and *X* remains unchanged^2, 3^; the constant *a* is the *Y* value at *X* = 1, which is often outside the range of the data being collected. Moreover, the value of *a* is changeable depending on the choice of units for *X* and *Y*; thus, this variable has received less attention because its significance is difficult to interpret. However, White and Gould^3^ emphasised the significance of the allometric intercept in ontogenetic and phylogenetic changes and as a taxonomic indicator. In recent, Halley^4^ pointed out that the allometric intercept represents prenatal brain mass at 1 gram of body mass and it corresponds to a timing where most mammalian fetuses transit from embryonic to fetal development.

The allometric relationships between morphometric traits and body mass have been investigated for various animal species. If the proportions of morphometric traits remain similar with increasing body size (geometric similarity), the linear dimensions should scale with the mass of the body to the power of 1/3. In addition, based on biophysics, McMahon^5^ proposed that for a quadruped animal to bear its own weight, the diameter and length of its trunk must be proportional to the mass of its body to the power of 0.375 and 0.25, respectively, considering an animal body as a circular cylinder (elastic similarity). For domestic bovids, chest girth (CG) is most frequently examined among morphometric traits because it is easily measurable and closely correlates with body weight (BW). If chest girth is considered as the product of trunk diameter and pi, the pattern of increase in chest girth is proportional to that of diameter. The allometric relationships between body weight and chest girth have been reported for several species and breeds (e.g., for four dairy cattle breeds by Brody *et al*.^6^; for Holstein-zebu crossbred heifers by Oliveira *et al*.^7^).

In morphology, three types of allometry have been defined depending on the target used for comparison^8^: ontogenetic, static and evolutionary. Ontogenetic allometry refers to the relationship between a trait and its size during the growth of an individual; static allometry indicates the relationship observed across individuals measured at similar developmental stages; and evolutionary allometry examines the relationship observed between the means for populations or species. Because the three levels of allometry are related and a higher level of allometry can be expressed as a function of allometry at lower levels, limited potential for evolution at a lower level would impose constraints at higher levels and consequently, constraints on phenotypic evolution^8^. A debated question in recent allometric research is whether invariance in allometry can be explained by stabilizing selection or by developmental or physiological constraints^9, 10^. Voje *et al*.^11^ found evidence that static allometry is evolvable and may not be an important constraint on a macroevolutionary time scale, but they obtained little evidence of microevolutionary changes in allometric slopes; these authors therefore concluded that the hypothesis of strongly constrained static allometric slopes remains viable. However, they had very little evidence of changes in ontogenetic allometries due to a lack of data. Although several studies have examined evolved responses in allometry under artificial selection^9, 12, 13^, they have focused on static allometry and not ontogenetic allometry.

The bovids experienced various forms of natural and artificial selection through adaptation to a diversity of environments before their domestication and subsequent breed improvement. In the present study, we statistically compared allometric parameters (slope and intercept) for various types of domestic bovids using cross-sectional ontogenetic data collected in the field. We then attempted to interpret the differences in the allometric parameters of chest girth to body weight among species and breeds in relation to their courses of differentiation, evolution and breed improvement to make inferences regarding the evolved responses in ontogenetic allometry to natural and artificial selection.

## Materials and Methods

### Data description

To compare allometric relationships among species, cross-sectional ontogenetic paired measurements of body weight and chest girth were collected for the following species and breeds of domestic bovids (the family Bovidae): cattle (*Bos taurus*), zebu-cattle (the hybrid of *Bos indicus* and *Bos taurus*), buffaloes (*Bubalus bubalis*), yaks (*Bos grunniens* or *Poephagus grunniens*) and goats (*Capra hircus*). Cattle, zebu-cattle, buffaloes and yaks belong to the subfamily Bovinae, whereas goats belong to Caprinae. There are two breeds each of zebu-cattle and buffaloes. Data for all animals were collected from conventional measurements at farms or the breeding center. The data collection was carried out in accord with the guidelines of the Kyoto University Animal Ethics Committee.

Descriptive statistics of the data sets used for the analyses are provided in Table 1. Only data from female animals were used in the analyses. The data set for each species/breed was collected at various stages of growth under field conditions; thus, information on precise age was lacking. The cattle (*Bos taurus*) data were collected between 1995–1996 and 2003–2005 on Mishima Island, Yamaguchi Prefecture, Japan. These data were collected from Mishima cattle; this breed is the oldest and only native breed among modern Japanese beef breeds (Wagyu) and was declared a “national natural treasure” in 1928. Because the breed has been isolated as a closed population on Mishima Island, the body shape and size of the ancestral indigenous Japanese cattle have been maintained^14^. The zebu-cattle and buffalo data were collected in 5 villages of the Chitwan District, Nepal, in 2001 and 2002. The zebu-cattle data included two crossbreeds: “Holstein-zebu” and “Jersey-zebu,” which are hybrids of Nepalese indigenous humped cattle (*Bos indicus*) and Western improved humpless dairy cattle (*Bos taurus*). For buffaloes, the Nepalese indigenous buffalo and its Murrah crossbreed were identified as the breeds of buffalo and referred to as “Nep. local buffalo” and “Murrah-cross buffalo,” respectively. Both breeds belong to the river type of buffalo, which have mainly been developed in the Indian subcontinent and used for milk production^15^. The yak data were collected in 2 villages in the Mustang District of Nepal in 2013 and 2014. Although the yak has been considered to be in the same genus as cattle (*Bos*), it has also sometimes been classified in its own genus (*Poephagus*). All of the yaks were purebred, i.e., not intercrossed with cattle. Zebu-cattle, buffalo and yak were regarded as a single population by species or breed, because the villages where the data were collected were located in the same areas and the rearing conditions were almost same. Data from goats born from 1997 to 2005 were collected at the Nagano Station of the National Livestock Breeding Center, Japan. The goat breed was Japanese-Saanen, which is the main breed in Japan that has been improved for milk production. The breed was established from the beginning to the middle of the 20th century through progressive crosses of Japanese indigenous and imported Saanen goats^16^.

**Table 1:** Descriptive statistics of the data sets used for the analyses.

	n.	Chest girth (cm)		Body weight (kg)	
		Mean ± SD	Min. − Max.	Mean ± SD	Min. − Max.
Cattle	712	137.3 ± 22.3	57.0–176.0	206.1 ± 76.8	17.5–440.0
Zebu-cattle					
Holstein-zebu	140	147.5 ± 14.3	109.0–183.0	265.8 ± 79.8	95.0–550.0
Jersey-zebu	66	143.0 ± 12.4	113.0–170.0	237.5 ± 62.7	96.0–348.0
Buffalo					
Murrah-cross buffalo	140	167.5 ± 19.6	76.0–197.0	339.3 ± 93.7	44.0–546.0
Nepalese local buffalo	195	167.1 ± 16.3	95.0–198.0	334.8 ± 83.8	74.5–514.0
Yak	68	133.7 ± 13.6	96.0–156.0	137.5 ± 36.6	56.5–199.0
Goat	395	78.9 ± 10.1	61.0–100.4	46.5 ± 17.0	21.9–85.8

### Statistical analyses

The bivariate allometry relating chest girth to body weight was analysed according to the allometric equation *Y* = *aX* ^*b*^, where *Y* = CG (cm), and *X* = BW (kg). Taking the natural logarithm of both sides, the equation can be linearly transformed: log*Y* = log(*a*) + *b*log*X*. All of the data were fitted in the equation at log scales via the ordinary least squares method using the PROC REG in SAS (SAS Institute Inc., Cary, NC, USA). Data points were considered as outliers and removed if the studentised residuals by species and breed were outside the range of −2.5 to 2.5^17^; 1.7% of the total data points were removed through this process. To assess the effect of species/breed on the parameters, we conducted meta-regression analysis by means of the following statistical model using PROC GLM in SAS, according to St-Pierre^18^:

$${Y}_{ij}={B}_{0}+{S}_{i}+{B}_{1}{X}_{ij}+{B}_{i}{X}_{ij}+{e}_{ij}$$

where *Y*_*ij*_ is the logarithm of the CG value of the *j*th data point in the *i*th species/breed, *X*_*ij*_ is the logarithm of the corresponding BW value, *B*_*0*_ is the overall intercept (log(*a*)) across the species/breeds, *S*_*i*_ is the effect of the *i*th species/breed on the intercept, *B*_*1*_ is the overall regression coefficient (*b*) across the species/breeds, *B*_*i*_ is the effect of the *i*th species/breed on the regression coefficient, and *e*_*ij*_ is the residual error. The significances of the comparisons of the regression coefficients (*B*_*i*_) between species/breeds were evaluated using the CONTRAST statement in SAS. The SAS statements used to produce the analysis according to the model are shown in the Supplementary Information.

Since species share many characteristics as a consequence of their common ancestry, phylogenetic comparative methods have been developed to control for the lack of statistical independence among species in the data^19^. On the other hand, the present statistical analyses ignored phylogenetic relationships due to lack of appropriate resources, and therefore there is the possibility that the differences across species and breeds might be overestimated.

### Evaluation of the intercept independent of the slope

**Figure 1:**
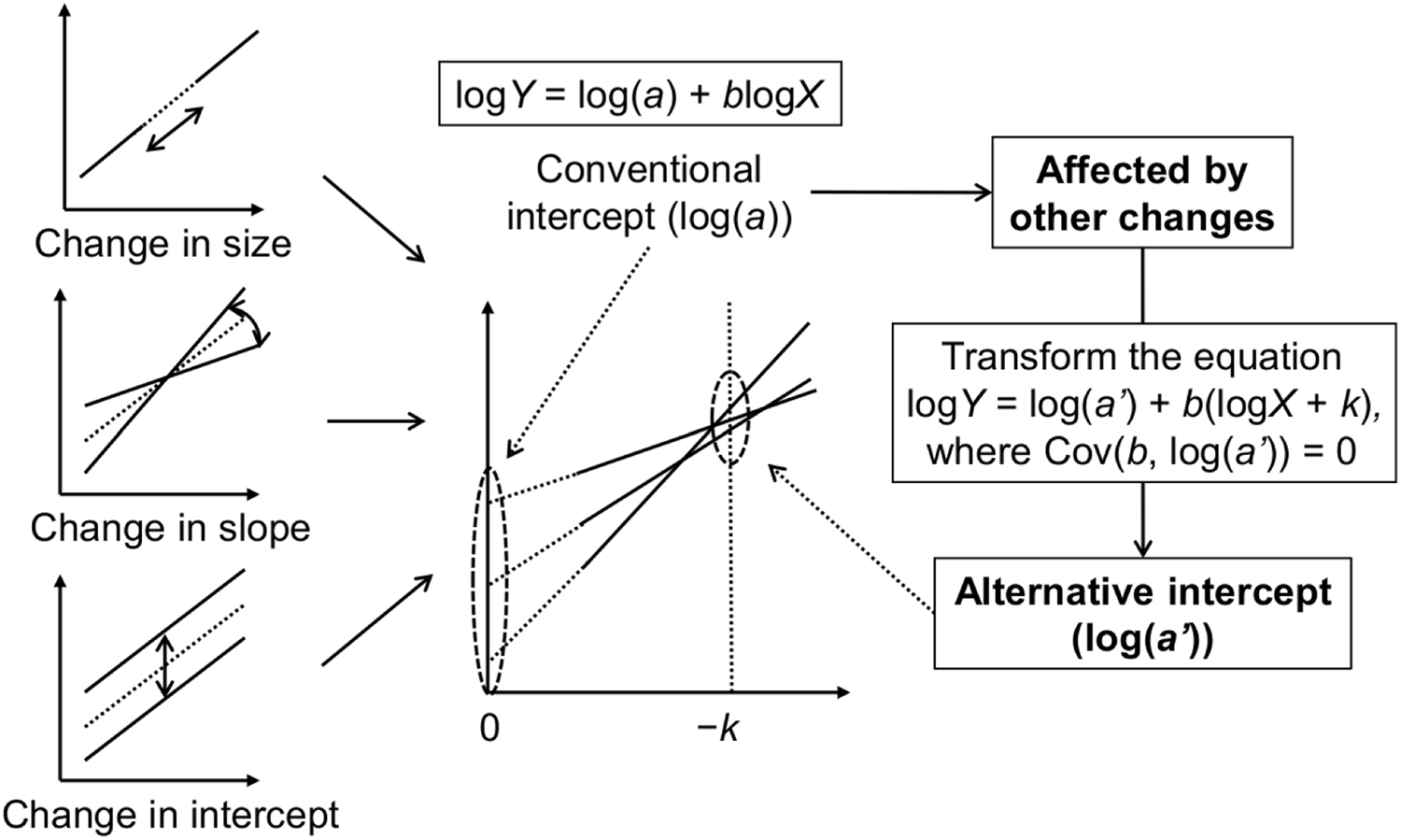
Conceptual description of the allometric intercept independent of the slope (the alternative intercept, log(*a’*)).

In past studies of allometry, inverse relationships between allometric slopes and intercepts have sometimes been found^3, 20^. Lumer^21^ showed that an inverse relationship would only hold when the allometric curves pass through a restricted area (that is, come close to intersecting) at a value of *X* greater than 1. Because the intercept in the equation can be changed by the choice of units, the correlation between the slope and intercept can be strong or weak and positive or negative. If the intersection occurs at *X* = 1, the correlation will disappear. Therefore, the value of the intercept can be affected by the value of the slope depending on the choice of units. White and Gould^3^ developed three classifications for the change in proportions that occurs in the evolution of organisms exhibiting allometric growth: (1) a simple size change with no alteration of the allometric constants, (2) a change in the slope, and (3) a change in the intercept independent of the slope. To detect changes in the intercept, Egset *et al*. evaluated the intercept at the population mean, to which they referred as ‘elevation’^12^. They explained that differences in elevation indicate differences in intercept when the allometric slope is constant across samples. However, because changes in the size, slope and intercept have been complex at macro time scales, we calculated the alternative intercept to examine differences in the intercept independent of the slope in the present study. The conceptual description of the intercept independent of the slope is shown in Fig. 1. For a linear relationship between *b* and log(*a*) across species and breeds, the relationship can be expressed as log(*a*) = *kb* + *l* + *ε*, where *k* and *l* are constants and *ε* is the residual error. Here, *l* + *ε* are independent of the explanatory variable *b*. Substituting into log*Y* = log(*a*) + *b*log*X*, the allometric equation can be expressed as log*Y* = (*l* + *ε*) + *b*(log*X* + *k*). To evaluate the intercepts independent of the slopes, an alternative intercept was calculated using the following equation: log*Y* = log(*a’*) + *b*(log*X* + *k*); hence, *Y* = *a’*(*X*/e^*−k*^)^*b*^, where *k* is the regression coefficient of the linear regression between log(*a*) and *b* (*k* = Cov(log(*a*), *b*)/Var(*b*)), *a’* is a new constant (log(*a’*) = *l + ε* = log(*a*) *− kb*) and *b* is equal to the slope in the original equation. The value of *a’* is the value of CG (cm) at BW = e^*−k*^ (kg) in the present study. Proof of the independence between *b* and log(*a’*) by mathematical expression is given in the Supplementary Information. Hereafter, we refer to log(*a’*) as ‘the alternative intercept’. The effect of species/breed on the alternative intercept was assessed using the same statistical model used for the original intercept.

### Data availability

The data analysed in the present study are available in the Supplementary Data.

## Results

**Figure 2:**
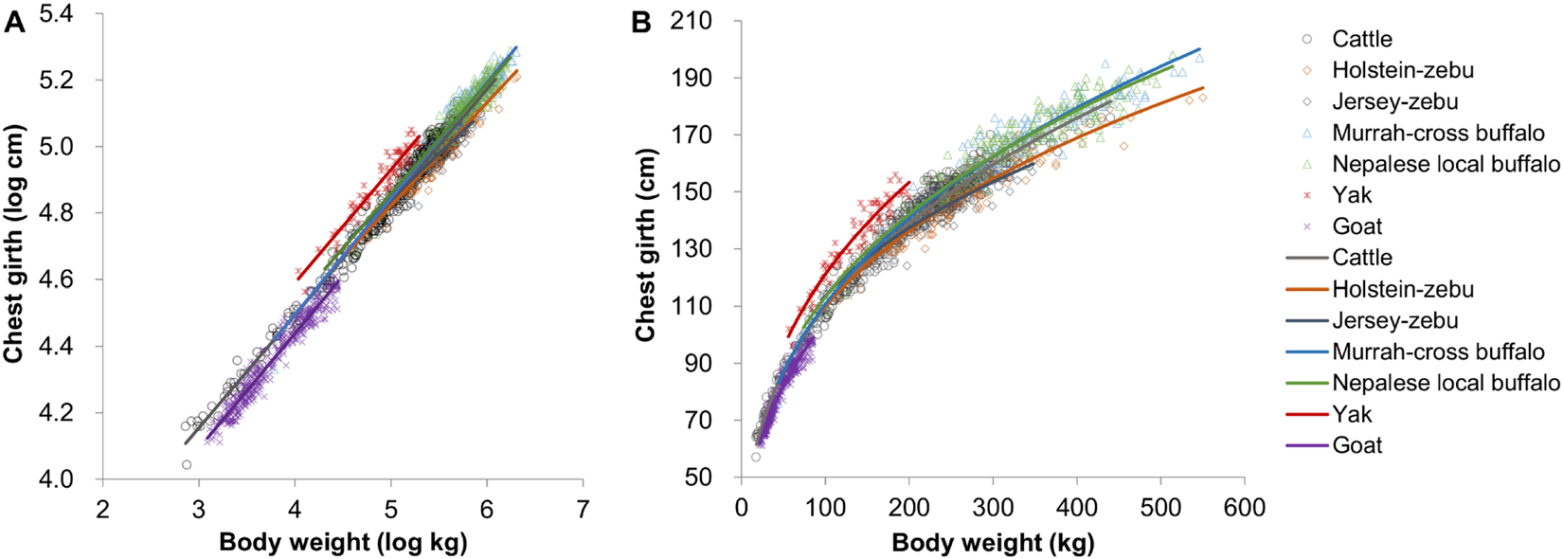
Estimated allometry with scatter plots of observations by species and breed. (**A**) Allometric lines on logarithmic scale. (**B**) Allometric curves on arithmetic scale.

The estimated allometric lines and curves of chest girth to body weight by species and breed are shown in Fig. 2, and the allometric parameters for the inter-species/breed comparisons are presented in Table 2. For all species and breeds, the coefficients of determination were high (>0.87). The mean slope for overall species/breeds was 0.3268, which was in closer agreement with the geometric similarity (0.333) than the elastic similarity (0.375), whereas the slope for Jersey-zebu was substantially lower than those for similarity. The allometric slopes differed between breeds within the same species (zebu-cattle and buffalo), whereas no significant differences were found among some species/breeds (e.g., among cattle, Murrah-cross buffalo, yak and goat).

**Table 2:** Estimated allometric parameters (parameter estimate  ±  s.e.) for chest girth (CG) relative to body weight (BW).

	Slope (*b*)	Intercept (log(*a*))	Alternative intercept (log(*a*’))	R^2^
Overall species/breeds	0.3268 ± 0.0030	3.211 ± 0.016	4.805 ± 0.002	—
*P-value*	<0.0001	<0.0001	<0.0001	
Cattle	0.3393 ± 0.0019^ab^	3.137 ± 0.010 ^cd^	4.792 ± 0.001^de^	0.98
Holstein-zebu	0.3102 ± 0.0080^c^	3.271 ± 0.044^b^	4.784 ± 0.006^e^	0.92
Jersey-zebu	0.2736 ± 0.0117^d^	3.473 ± 0.063^a^	4.808 ± 0.007^bc^	0.87
Murrah-cross buffalo	0.3487 ± 0.0061^a^	3.101 ± 0.035^de^	4.802 ± 0.006 ^cd^	0.96
Nepalese local buffalo	0.3294 ± 0.0065^bc^	3.212 ± 0.037^bc^	4.819 ± 0.006^b^	0.92
Yak	0.3410 ± 0.0116^ab^	3.226 ± 0.057^bcd^	4.889 ± 0.003^a^	0.93
Goat	0.3457 ± 0.0040^a^	3.056 ± 0.015^e^	4.743 ± 0.005 ^f^	0.95

R^2^; coefficient of determination. (a,b,c,d,e,f) Values in the same columns in the interspecies/breeds comparison with different superscripts differ significantly (*P* < 0.05). The slopes and intercepts were estimated from the equation log*CG* = log(*a*) + *b*log*BW*. The alternative intercepts were estimated from the equation log*CG* = log(*a’*) + *b*(log*BW* + *k*). The value *k* = −4.8791 was used. *P-value* indicating whether or not the slope or intercept is affected by species or breed.

**Figure 3:**
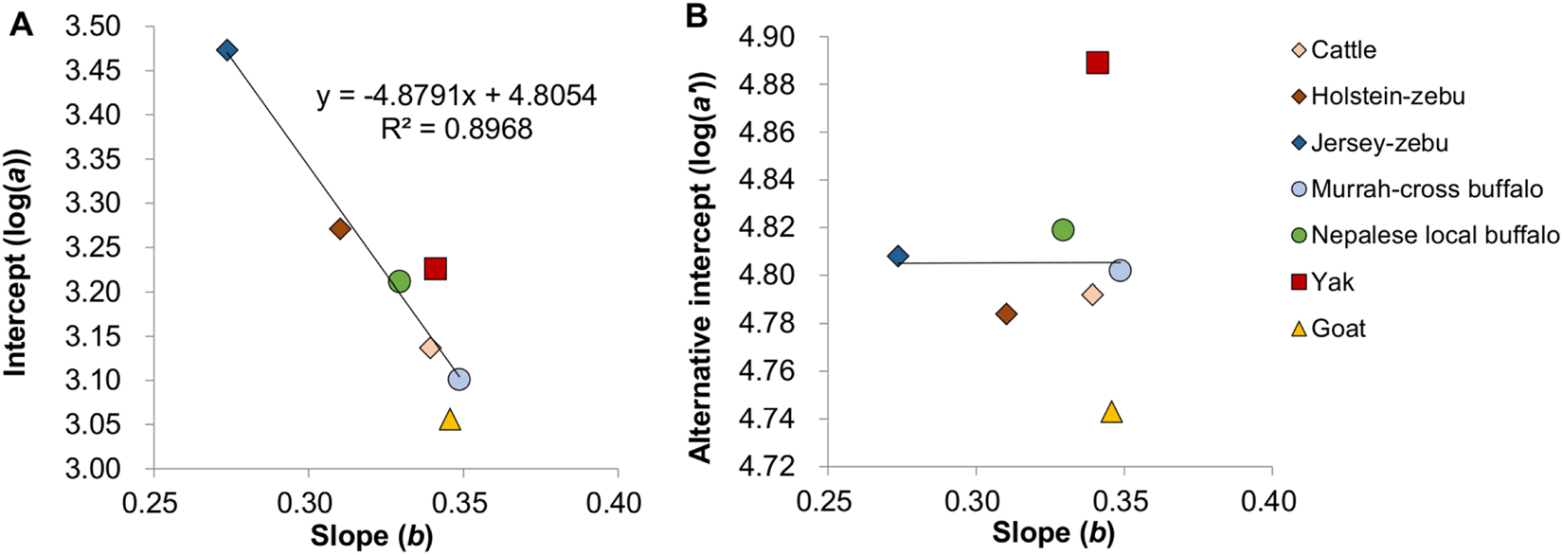
Scatter plots of allometric parameters across species and breeds. (**A**) Slopes (*b*) and intercepts (log(*a*)). (**B**) Slopes and alternative intercepts (log(*a’*)).

Scatter plots of allometric slopes and intercepts across species and breeds are shown in Fig. 3A. A strong negative correlation (*r* = −0.95) was observed between slope and intercept across the species and breeds, indicating that the intercepts were strongly affected by the slopes. From the regression coefficient between slope and intercept, *k* = −4.8791 was obtained, which indicates that the allometric lines come close to intersecting at BW = 131.5 kg. The alternative intercept was 4.805 in the mean and was uncorrelated with slope (Fig. 3B). The alternative intercepts of yaks and goats were significantly higher and lower than those of all of the other species, respectively, whereas those of the other species and breeds were quite similar and, in some cases, not significantly different (Table 2).

## Discussion

The species of bovids examined in the present study diverged millions of years ago and have sizes and shapes that have adapted to the environment in different ways under natural selection. From the viewpoint of molecular phylogeny, goats are considered to have diverged from bovines approximately 18.9 million years ago (Ma); buffaloes diverged from the other bovine approximately 14.3 Ma^22^; yaks are estimated to have diverged from cattle and zebu approximately 4.9 Ma^23^; and cattle and zebu diverged approximately 0.1 Ma^22^. From their domestication to the present, these animals have been continuously and strongly selected by humans for the purpose of improving production traits. This artificial selection has led to changes in the sizes and shapes of these animals; for instance, selection for the improvement of meat yield has increased the proportion of lean meat, and the development of dairy breeds has led to much higher lactation than is required by calves and to large udders.

Our present results showed that the allometric slopes were constant across different species (e.g., cattle, Murrah-cross buffalo, yak and goat), which appears to indicate that the relative growth rate of chest girth to body weight (i.e., slope) is constrained by developmental mechanisms even on macro time scales. Constant allometric slopes have been interpreted as the result of invariant growth regulation mechanisms (allometric constraint hypothesis). This hypothesis postulates that static and ontogenetic allometric slope remains stable at macro-evolutional time scales because of the evolutionary restriction in trajectories imposed by the allometries^24^. Tsuboi *et al*.^25^ found that brain-body static allometric slope may represent an evolutionary constraint in Lake Tanganyika cichlids, and Firmat *et al*.^24^ found that the macroevolutionary divergence of molar traits for fossil rodents be constrained by static allometric relationships. The present results on ontogenetic allometries are consistent with the findings of these previous studies on static allometries. Nevertheless, the slopes differed between breeds in zebu-cattle and buffaloes despite the fact that breed improvement has a far shorter history (at most several hundred years) than the differentiation and evolution of species. The result is apparently inconsistent with the indication that allometric slopes are constrained. Experiments on artificial selection of wing shape or wing-body size scaling in fruit flies demonstrated that the static allometric slope could be altered within several generations^9, 13^ but that the response was rapidly lost when selection was suspended^9^. Furthermore, they demonstrated that the response was erratic in contrast to that of the intercept, which responded smoothly and rapidly to selection^13^. Bolstad *et al*.^9^ explained that the evolutionary change in the allometric slope resulted in deleterious pleiotropic responses in other aspects of the phenotype and consequently induced countervailing natural selection to return the slope to its initial value. Although the type of allometry, species and traits in the present study are different from those examined in these previous studies, the differences in slope found in the present study may also be the result of specific artificial selection. For instance, the differences in allometric slope between the breeds of zebu-cattle might be attributed to breed improvement for dairy production, which is related to udder morphology. The udder size of bovids is correlated with milk yield^26^, and udder growth is affected by estrogen and growth hormone during puberty^27^. Since the growth rate of the udders may affect that of body weight but not that of chest girth, the different growth rates of the udders among dairy breeds might indirectly generate differences in the allometric slope. In response to the study by Bolstad *et al*.^9^, Harrison^10^ commented that it is certainly conceivable that the organ-specific variation in responsiveness to growth-promoting neuroendocrine signals is heritable and could provide a mechanism allowing variation in allometric slopes across populations and species. Although the mechanism underlying the alteration of the allometric slope cannot be explained by the present results, it might be possible that the slopes were altered through specific artificial selection and that the differences between breeds within species have been maintained by the continuous selection.

**Figure 4:**
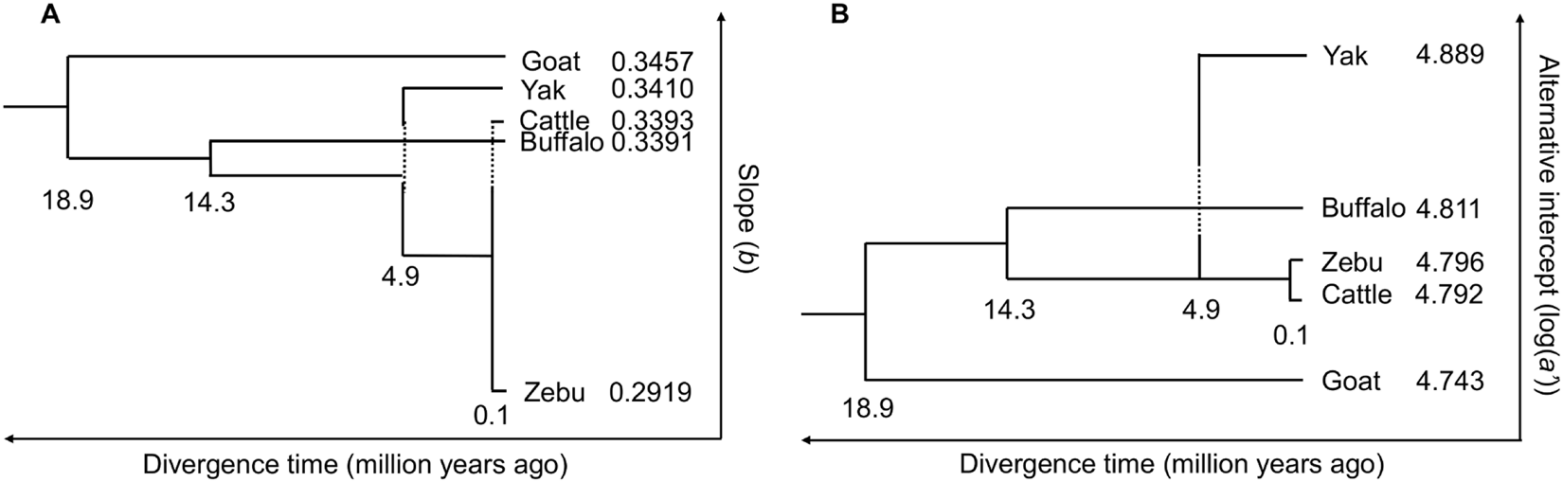
Relationships between allometric parameters and species divergence time. (**A**) Slopes (*b*) and divergence time. (**B**) Alternative intercepts (log(*a’*)) and divergence time. For buffalo and zebu, the mean values of the two breeds of each species are shown. The numbers under the branch points indicate divergence times (millions of years ago), which were derived from analyses of molecular phylogeny^22, 23^.

In the present analysis, the original intercepts (log(*a*)) were strongly affected by the slopes. In contrast, the alternative intercept can ensure independence from the slopes. Hereinafter, we focus on interpreting the differences in the alternative intercept (log(*a’*)) (also see the evaluation of three different intercept-related allometric parameters in the Supplementary Information). Since the alternative intercept was uncorrelated with slope, a fair comparison of the intrinsic difference in allometric intercept (i.e., proportional shift in the size of *Y* relative to *X*) was possible. The similar alternative intercepts between the breeds in zebu-cattle and in buffaloes indicate that the alternative intercepts of chest girth to body weight are difficult to alter on shorter time scales. Because of this difficulty, the observed differences in the alternative intercept among species can be explained by the morphological phylogenetic changes occurring on macro time scales. Figure 4 shows the relationships between allometric parameters and species divergence time. With the exception of yak, the differences in the alternative intercept among species might better correspond to their divergence times than the differences in allometric slope. This relationship was even applicable to goats, which differ substantially from the other species in both size and taxonomy. Among the examined species, yaks are distinguished by a particularly high intercept, although yaks are sufficiently closely related to cattle for intercrossing to occur. This high alternative intercept can be explained by the unique morphological evolution of yaks. Yaks have acquired larger lungs and hearts relative to their overall body size and have developed 14 to 15 pairs of thoracic ribs as an adaptation to the low oxygen content of the air in high mountainous regions^28^. It is therefore reasonable that the chest girth of yaks relative to their body weight was consistently higher than in the other species, as shown in Fig. 1. Bolstad *et al*.^9^ reported that the static allometric intercept could be altered and that any alterations were largely stable after selection ceased, in contrast to the case for the slope. The present study found that there was little alteration of the alternative intercept over short time scales and that differences in the alternative intercept among species have been maintained over macro time scales. It should be noted that because the alternative intercept is derived from a purely mathematical point, the value itself do not necessarily have consistent biological meanings. However, the interspecific differences in the alternative intercept may reflect traces of the morphological phylogenetic changes derived from differentiation and evolution over millions of years.

## Conclusion

Our comparative analysis of allometry between body weight and chest girth across species and breeds of domestic bovids suggest the following findings regarding the evolved responses of ontogenetic allometry: the allometric slope is typically invariant among species but can be changed under strong, specific selection, and the intercept independent of the slope is difficult to alter over a short time; therefore, the differences in this intercept can reflect phylogenetic changes that have occurred over their differentiation and evolution. These implications will be helpful for obtaining a deeper understanding of evolved responses in allometry.

## Electronic supplementary material

Supplementary Information

Supplementary Data
