# Supplementary material for: Interspecific comparison of allometry between body weight and chest girth in domestic bovids
Source: Sci Rep. 2017 Jul 6;7:4817. doi: 10.1038/s41598-017-04976-z (PMC5500593; doi:10.1038/s41598-017-04976-z)
Supplement: Supplementary file 1 — Supplementary Information [file 41598_2017_4976_MOESM1_ESM.pdf]

Supplementary Information for

Interspecific comparison of allometry between body weight and chest girth in domestic bovids

Hiroki Anzai[1*], Kazato Oishi[1], Hajime Kumagai[1], Eiji Hosoi[2], Yoshitaka Nakanishi[3], and Hiroyuki Hirooka[1]

1. Graduate School of Agriculture, Kyoto University, Kyoto, 606-8502, Japan
2. Faculty of Agriculture, Yamaguchi University, Yamaguchi, 753-8515, Japan
3. Faculty of Agriculture, Kagoshima University, Kagoshima, 890-8580, Japan

[*]e-mail: anzai@kais.kyoto-u.ac.jp

SAS statements for meta-regression analysis

The SAS statements used to produce the analysis according to the model described in the manuscript are as follows:

```
PROC GLM;
CLASS Breed;
MODEL Lcg = Breed Lbw Lbw*Breed;
LSMEANS Breed /at Lbw=0 STDERR PDIFF;
ESTIMATE 'Overall intercept' INTERCEPT 7 Breed 1 1 1 1 1 1 1/DIVISOR=7;
ESTIMATE 'Overall slope' Lbw 7 Lbw*Breed 1 1 1 1 1 1 1/DIVSOR=7;
ESTIMATE 'Slope 1' Lbw 1 Lbw*Breed 1;
ESTIMATE 'Slope 2' Lbw 1 Lbw*Breed 0 1;
ESTIMATE 'Slope 3' Lbw 1 Lbw*Breed 0 0 1;
ESTIMATE 'Slope 4' Lbw 1 Lbw*Breed 0 0 0 1;
ESTIMATE 'Slope 5' Lbw 1 Lbw*Breed 0 0 0 0 1;
ESTIMATE 'Slope 6' Lbw 1 Lbw*Breed 0 0 0 0 0 1;
ESTIMATE 'Slope 7' Lbw 1 Lbw*Breed 0 0 0 0 0 0 1;
CONTRAST 'Slope 1 vs 2' Lbw 0 Lbw*Breed 1 -1 0 0 0 0 0;
CONTRAST 'Slope 1 vs 3' Lbw 0 Lbw*Breed 1 0 -1 0 0 0 0;
CONTRAST 'Slope 1 vs 4' Lbw 0 Lbw*Breed 1 0 0 -1 0 0 0;
CONTRAST 'Slope 1 vs 5' Lbw 0 Lbw*Breed 1 0 0 0 -1 0 0;
```

```
CONTRAST 'Slope 1 vs 6' Lbw 0 Lbw*Breed 1 0 0 0 0 -1 0;
CONTRAST 'Slope 1 vs 7' Lbw 0 Lbw*Breed 1 0 0 0 0 0 -1;
CONTRAST 'Slope 2 vs 3' Lbw 0 Lbw*Breed 0 1 -1 0 0 0 0;
CONTRAST 'Slope 2 vs 4' Lbw 0 Lbw*Breed 0 1 0 -1 0 0 0;
CONTRAST 'Slope 2 vs 5' Lbw 0 Lbw*Breed 0 1 0 0 -1 0 0;
CONTRAST 'Slope 2 vs 6' Lbw 0 Lbw*Breed 0 1 0 0 0 -1 0;
CONTRAST 'Slope 2 vs 7' Lbw 0 Lbw*Breed 0 1 0 0 0 0 -1;
CONTRAST 'Slope 3 vs 4' Lbw 0 Lbw*Breed 0 0 1 -1 0 0 0;
CONTRAST 'Slope 3 vs 5' Lbw 0 Lbw*Breed 0 0 1 0 -1 0 0;
CONTRAST 'Slope 3 vs 6' Lbw 0 Lbw*Breed 0 0 1 0 0 -1 0;
CONTRAST 'Slope 3 vs 7' Lbw 0 Lbw*Breed 0 0 1 0 0 0 -1;
CONTRAST 'Slope 4 vs 5' Lbw 0 Lbw*Breed 0 0 0 1 -1 0 0;
CONTRAST 'Slope 4 vs 6' Lbw 0 Lbw*Breed 0 0 0 1 0 -1 0;
CONTRAST 'Slope 4 vs 7' Lbw 0 Lbw*Breed 0 0 0 1 0 0 -1;
CONTRAST 'Slope 5 vs 6' Lbw 0 Lbw*Breed 0 0 0 0 1 -1 0;
CONTRAST 'Slope 5 vs 7' Lbw 0 Lbw*Breed 0 0 0 0 1 0 -1;
CONTRAST 'Slope 6 vs 7' Lbw 0 Lbw*Breed 0 0 0 0 0 1 -1;
RUN;
```

The variables 'Lcg' and 'Lbw' indicate log(CG) and log(BW), respectively. The variable 'Breed' indicates seven species/breeds (i.e., cattle, Holstein-zebu, Jersey-zebu, Murrah-cross buffalo, Nepalese local buffalo, yak and goat). The fixed effect 'Breed' and the interaction effect 'Lbw*Breed' represent the effect of the species/breed on the intercept and slope in the allometric equation, respectively.

Proof of the independence of b and log(a')

To derive the intercept independent of the allometric slope (b), the allometric equation ($\log Y = \log(a) + b\log X$) is transformed as $\log Y = \log(a') + b(\log X + k)$, where $\log(a')$ is the alternative intercept and k is the constant (see text).

By using the relation

$\log Y = \log(a) + b\log X = \log(a') + b(\log X + k)$,

thus $\log(a') = \log(a) - kb$,

and the formula for constants A and B and variables x and y:

$$\mathrm{Var}(Ax + By) = A^2{*}\mathrm{Var}(x) + B^2{*}\mathrm{Var}(y) + 2AB{*}\mathrm{Cov}(x, y),$$

we can derive the following equality:

$2\mathrm{Cov}(\log(a'), b) = \mathrm{Var}(\log(a) - kb + b) - \mathrm{Var}(\log(a) - kb) - \mathrm{Var}(b)$

$= \mathrm{Var}(\log(a) + b(1 - k)\ ) - \mathrm{Var}(\log(a) - kb) - \mathrm{Var}(b)$

$= \mathrm{Var}(\log(a) + (1 - k)^2\mathrm{Var}(b) + 2(1 - k)\mathrm{Cov}(\log(a), b)$

$- (\mathrm{Var}(\log(a) + k^2\mathrm{Var}(b) - 2k\mathrm{Cov}(\log(a), b)) - \mathrm{Var}(b)$

$= -2k\mathrm{Var}(b) + 2\mathrm{Cov}(\log(a), b)$.

Thus $\mathrm{Cov}(\log(a'), b) = -k\mathrm{Var}(b) + \mathrm{Cov}(\log(a), b)$.

This equality implies that $\mathrm{Cov}(\log(a'), b) = 0$ when $k = \mathrm{Cov}(\log(a), b)/\mathrm{Var}(b)$, which is the regression coefficient of the linear regression between $\log(a)$ and b.

Evaluation of three different intercept-related parameters

In the present study, the original intercept (log(*a*)) and the alternative intercept (log(*a'*)) were used for interspecific comparison of body weight and chest girth in domestic bovids. On the other hand, Egset *et al.*[12] used a different intercept related parameter, to which they referred as 'elevation'. These three parameters are the predicted log$Y$ values of the allometric regression at certain X values; the original intercept is the value at log$X$ = 0 (i.e., X = 1), the alternative intercept is the value at log$X$ = $-k$ (k is the regression coefficient between the allometric slopes and the original intercept), and the elevation is the value at X = the population mean.

These three different intercept-related parameters estimated from the present data are shown in Table S1. The population mean of the data was 5.013 (BW = 150.4 kg). For the analysis with the present data, the alternative intercept and the elevation were obtained at close points on x-axis, thus the two parameters had similar values, but were far different from the original intercepts. It should be however noted that these two parameters are not necessarily obtained at close points on x-axis, because the procedures for calculation are different.

There are different implications between the elevation and the alternative intercept. The elevation can be interpreted as the relative size of trait at population mean size, because natural selection may target mean body size, but it does not ensure independence between slopes and intercepts. Gould[20] pointed out that for allometric regressions differing in slope, an unambiguous biological interpretation for intercept will not be available until methods are devised to separate out the variation in intercepts due to differences in slopes. The alternative intercept can be used to discover implications of intercept, because the alternative intercepts across samples are certainly independent of slopes. Evolutionary changes in allometry caused by changes in the intercept independent of the slope illustrated by White and Gould[3] may be explained through the alternative intercept.

	Intercept (log(a))	Alternative intercept (log(a'))	Elevation (log(a''))
Overall species/breeds	3.211 ± 0.016	4.805 ± 0.002	4.849 ± 0.002
Cattle	3.137 ± 0.010[cd]	4.792 ± 0.001[de]	4.838 ± 0.001[d]
Holstein-zebu	3.271 ± 0.044[b]	4.784 ± 0.006[e]	4.826 ± 0.005[e]
Jersey-zebu	3.473 ± 0.063[a]	4.808 ± 0.007[bc]	4.845 ± 0.006[cd]
Murrah-cross buffalo	3.101 ± 0.035[de]	4.802 ± 0.006[cd]	4.849 ± 0.005[b]
Nepalese local buffalo	3.212 ± 0.037[bc]	4.819 ± 0.006[b]	4.863 ± 0.005[bc]
Yak	3.226 ± 0.057[bcd]	4.889 ± 0.003[a]	4.935 ± 0.004[a]
Goat	3.056 ± 0.015[e]	4.743 ± 0.005[f]	4.789 ± 0.005[f]
Correlation coefficient with slope	-0.97	0.00	0.08

Table S1. Estimated three different intercept-related parameters (parameter estimate ± s.e.). (a,b,c,d,e,f) Values in the same columns in the interspecies/breeds comparison with different superscripts differ significantly ($P < 0.05$). The slopes and intercepts were estimated from the equation $\log CG = \log(a) + b\log BW$. The alternative intercepts were estimated from the equation $\log CG = \log(a') + b(\log BW + k)$. The value $k = -4.8791$ was used. The elevations were estimated from the equation $\log CG = \log(a'') + b(\log BW - \text{(population mean)})$. The population mean of the data was 5.013 (BW = 150.4 kg).
